# Masticatory Efficiency in Orthodontic Patients with Craniofacial Disorder

**DOI:** 10.3390/ijerph20054324

**Published:** 2023-02-28

**Authors:** Maria Schmidt, Gregor Slavicek, Florian Slavicek, Matthias C. Schulz, Maite Aretxabaleta, Josephine Effert, Bernd Koos, Christina Weise

**Affiliations:** 1 Department of Orthodontics, University Hospital Tuebingen, Osianderstr. 2-8, 72076 Tuebingen, Germany; 2 Orehab Minds GmbH, Zettachring 2, 70567 Stuttgart, Germany; 3 Department of Oral and Maxillofacial Surgery, University Hospital Tuebingen, Osianderstr. 2-8, 72076 Tuebingen, Germany

**Keywords:** craniofacial malformation, standard food model test, cleft lip and palate, robin sequence, mastication, chewing, orthodontic patients, birth defect, sieve method, stomatognathic system

## Abstract

This study evaluates the masticatory efficiency in patients with craniofacial disorders (CD) compared to controls (C). A total of 119 participants (7–21 years), divided into CD group (n = 42, mean age 13.45 ± 5.2 years) and C group (n = 77, mean age 14.3 ± 3.27 years) under an orthodontic treatment were included. Masticatory efficiency was assessed using a standard food model test. The masticated food was examined according to its number of particles (n) and area (mm^2^), wherein a higher number of particles alongside a smaller area was an indication of better masticatory efficiency. Additionally, the influence of cleft formation, chewing side, dentition stage, age and sex were evaluated. Patients with CD chewed the standardized food in fewer particles (n_CD_ = 61.76 vs. n_C_ = 84.58), with a significantly higher amount of area than the controls (A_CD_ = 192.91 mm^2^ vs. A_C_ = 146.84 mm^2^; *p* = 0.04). In conclusion, patients with CD showed a significantly decreased mastication efficiency compared to healthy patients. Factors such as stage of cleft formation, chewing side, dentition stage and age showed an influence on masticatory efficiency, whereas no gender effect on the masticatory efficiency of CD patients was found.

## 1. Introduction

Within craniofacial disorders (CD), cleft lip and/or palate (CL/P) is the most commonly occurring disorder, with a prevalence of 1:600 [1]. This malformation varies in the degrees of visibility and severity of the cleft formation, in which different combinations can be distinguished: left or right sided unilateral cleft, bilateral CL/P, cleft lip with or without alveolus (CL) and a cleft palate only (CP). The latter can be found in 80–90% of the cases of patients with Robin sequence (RS), where a prevalence of 11.3:100,000 can be expected [2]. This congenital disorder is characterized by the triad of mandibular retrognathia, glossoptosis and resultant upper airway obstructions [3]. Both patients with CL/P and RS exhibit severe physical and functional difficulties in the stomatologic system. Therefore, rehabilitation therapy that includes an interdisciplinary team is necessary, in which the duration and intensity needed to improve the functional parameters depends on the severity of the CD.

Mastication function is one of the most important physical roles of the craniomaxillofacial system; it is understood as the amount of chewing action or the ability to break down and process food, with the goal of forming a food bolus that will be subsequently swallowed [4]. Whereas the term “masticatory function” has been used for both subjective and objective measurements in the literature, “masticatory efficiency” expresses the objective aspects of mastication and is commonly used in clinical tests. It is defined as the effort required to achieve a normal degree of comminution of food with standardized testing settings [5]. Several validated measurement methods have already been described in the literature to quantify and evaluate masticatory performance [6,7,8,9,10,11]. One of the first used methods was the so-called “sieve method”, where the assessment of mastication efficiency consisted of a manual evaluation of the average particle number and size of previously masticated model food [12,13,14,15,16]. Nowadays, this has been replaced by newer methods that are based on the optical scanning of chewed particles and the calculation of average parameters by software [4,10,17].

The influence of craniofacial and occlusal morphology on masticatory efficiency has been extensively described for healthy populations in the literature [18,19,20,21,22,23]. Arch form deformation, jaw discrepancies in sagittal and transversal dimensions, as well as scar formation after surgery due to the CD, are known to have a negative influence on masticatory efficiency [24]. Therefore, any performed orthodontic treatment should aim to achieve a masticatory efficiency comparable to that of healthy individuals. Moreover, understanding the influence of an occurring malformation which affects both dentofacial and skeletofacial morphology and thus, masticatory efficiency might have a clinical relevance that needs to be accounted for in the comprehensive management of interdisciplinary therapy. Knowledge of the alterations in the stomatognathic system and the differences from healthy patients could be the key to improving therapy for a good functional rehabilitation result. Despite this, the literature in this field is scarce, and the authors are unaware of any study evaluating the masticatory efficiency of orthodontic patients affected by CD.

In this scenario, this study aims to evaluate the masticatory efficiency of patients with congenital CD during an orthodontic treatment, as well as comparing it to healthy patients. In addition, the influence of different parameters such as, sex, cleft formation and dentition stage on chewing ability was also assessed. In this scenario, different null hypotheses were defined for this study:

- Patients with CD show no difference in masticatory efficiency compared to healthy patients without CD.
- The different hardness levels of the food samples do not significantly affect the masticatory efficiency of patients with CD.
- The different CDs and cleft formations do not differ in masticatory efficiency.
- There are no differences in the chewing side masticatory efficiency for the different CD cleft formations.
- Other secondary factors, such as stages of dentition, age and sex, do not significantly affect the masticatory efficiency of patients with CD.

## 2. Materials and Methods

### 2.1. Study Design

This cross-sectional study was designed to be prospective and monocentric at the Department of Orthodontics at Tübingen University Hospital. During a routine follow-up visit, patients were invited to participate by the clinician that was directly involved in their orthodontic care. All patients and their legal guardians were informed both verbally and in writing about the different aspects of the study: voluntary participation, the procedure to be performed, the aim of the study and the pseudonymization of the collected data. A consent form for each participant was signed by at least one parent or caregiver prior to data collection. All examinations could be carried out in one session of approximately 20 min, and were non-invasive as well as non-stressful for the participants. This study was approved by the institutional ethics committee of Tübingen University Hospital (Germany) (approval number: 188/2019BO1).

### 2.2. Participants

Patients receiving an orthodontic treatment in the Department of Orthodontics at Tübingen University Hospital (Germany) were selected by the treating orthodontist. Examinations were performed between 08/2019 and 08/2021, yielding a total of 140 patients that fulfilled the inclusion criteria. Nine patients were not interested in the study, yielding a final study population of 131 patients aged between 7 and 21 years. Due to COVID-19 pandemic restrictions, there was a break of three months in the examination period. The following inclusion criteria were applied:

- CD group: All variations of CL/P and RS.
- Current orthodontic treatment in the Department of Orthodontics at Tübingen University Hospital, Germany.
- Control group (C): no CD. The diagnosis was confirmed by the clinical picture and record.
- Age between 7–21 years. Patients were divided into age groups of either 7–12 or 13–21 years. 12 years was employed as division point, as permanent dentition should be completed by then, and also because an age-related change in mastication can be expected [25].

Exclusion criteria were defined as follows:

- CD group: additional complex CD (syndromes), psychological limitations, general illnesses and non-mastery of the German language.
- Patients younger than 7 and older than 21 years. Patients younger than 7 years were excluded due to the fact that only at about 6 years does the permanent teeth emergence start, and also that the molars might start to erupt but do not have an occlusal relationship; this might influence masticatory efficiency. Patients older than 21 were excluded, considering that these patients should have completed tooth development (i.e., a 3rd molar erupted) and facial growth.

### 2.3. Standardized Food Model Test

For the evaluation of masticatory efficiency, a standardized fragmentation method based on a standardized chewing test unit with the automated food model test Occlusal Systems CHEW (Orehab minds GmbH, Stuttgart, Germany) was employed. It was first described and invented by Slavicek et al. in 2009 [26]. This test system consists of round elastic food test units, calibrated recording plates, a sieve and a metal box with a fixed digital camera for the standardized photographing of the test plate. The employed test bodies are composed of comestible gelatins (Gelatin SPM 5765, Biogel AG, Luzern, Switzerland) that were prepared in three different hardness levels to simulate the total mass of standard or conventional food. The gelatin to water ratio was modified, and the achieved hardness level was differentiated by color addition (green as soft = 15.5 g per mass, yellow as medium = 23 g per mass, red as hard = 31 g per mass). All food bodies were identically flavored and prepared in the same cylindrical shape (ø = 2 cm, h = 1 cm). A standard protocol was used for every patient, where every participant was instructed to chew the test food into as many pieces as possible without swallowing them. For every hardness level, three different cycle variations were performed: chewing only on the right side, chewing only on the left side and chewing on both sides. Thus, all patients underwent nine cycles, each cycle lasting 30 s. The chewed food was collected at the end of each chewing cycle into a sieve, rinsed with cold water and spread on the test plate. Special care was taken to ensure that all particles were easily identifiable and represented as individual parts on the slide. Then, each test plate was processed by a standardized photographing unit, and the obtained images were transferred to Orehab Minds GmbH for further processing. The company calculated the number (n) and area (mm^2^) of the particles by means of a computer-aided analysis.

### 2.4. Statistical Data Analyses

Patient data were collected from clinical records and pseudonymized. Statistical evaluation and descriptive statistics of the data were performed using JMP (Version 15.2.0, SAS Institute Inc., Cary, NC, USA). Descriptive statistics included means and standard deviations (SD) of each sample. For normally distributed data, a *t*-test and ANOVA were applied. Statistical significance was considered at *p* < 0.05. Masticatory efficiency differences between groups were statistically evaluated alongside other secondary factors such as gender, age and chewing side. The effect of the dentition stage was only analyzed in a descriptive way, as the sample size was too small. Within the CD group, differences among the cleft formations in masticatory efficiency were analyzed, as well as specific differences in CD and employed chewing sides.

## 3. Results

### 3.1. Characteristics of the Study Participants

This study included a total of 119 orthodontic patients (Table 1), which included 42 participants with CD: 38 CL/P and 4 RS patients. CL/P patients were further classified into right-sided cleft (n = 8), left-sided cleft (n = 18), bilateral cleft (n = 6), cleft lip (n = 2) and cleft palate (n = 4). Some 77 healthy patients with no CD were also considered in this study. The mean age of participants was 13.45 years in CD patients, and 14.3 years in the healthy participants. In addition, patients were classified according to the age in two groups of either 7–12 or 13–21 years. The sex ratio was equally distributed in both groups. Finally, patients were also classified with respect to their dentition stage (Table 1).

### 3.2. Masticatory Efficiency

**Figure 1:**
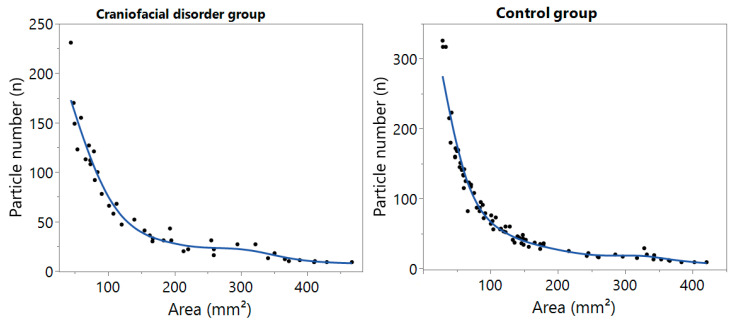
Masticatory efficiency curves comparing patients with craniofacial disorders (n = 42) and the healthy control group (n = 77). The obtained chewed particle number (n) is plotted against the obtained surface area (mm^2^).

The overall test (all nine chewing cycles) yielded a statistical significance in masticatory efficiency values between patients with CD and without, with a lower masticatory efficiency in patients with CD. This difference is shown in Figure 1 and Table 2. In average, healthy participants masticated the test bodies in significantly smaller areas (A_C_ = 146.84 mm^2^) than those obtained in patients with CD (A_CD_ = 192.91 mm^2^, *p* = 0.04). Despite this, no statistically significance was found in the number of chewed particles between the CD (n_CD_ = 61.76) and control group (n_C_ = 84.58).

More detailed information was obtained by differentiating the obtained results by chewing side and employed sample body hardness (Table 2). Patients with CD showed decreased average numbers of particles and an increased amount of area in all different combinations of chewing side and test body hardness in comparison to the healthy group. All of those differences were statistically not significant, except the left-sided chewing process, as well as medium to hard samples. A significant decreased left-side masticatory efficiency was observed for patients with CD, shown by the decrease in particle number (n_CD_ = 20.17 vs. n_C_ = 30.69; *p* = 0.02), and the increase in area (A_CD_ = 215.40 mm^2^ vs. A_C_ = 145.40 mm^2^; *p* > 0.00) which were significantly different to the values observed in the group without a malformation. Moreover, the CD group had a significantly lower number of particles in hard test bodies than the control group (n_CD_ = 9.21 vs. n_C_ = 29.40; *p* = 0.04), and a significantly higher area in the medium level (A_CD_ = 209.63 mm^2^ vs. A_C_ = 157.52 mm^2^; *p* = 0.04).

**Figure 2:**
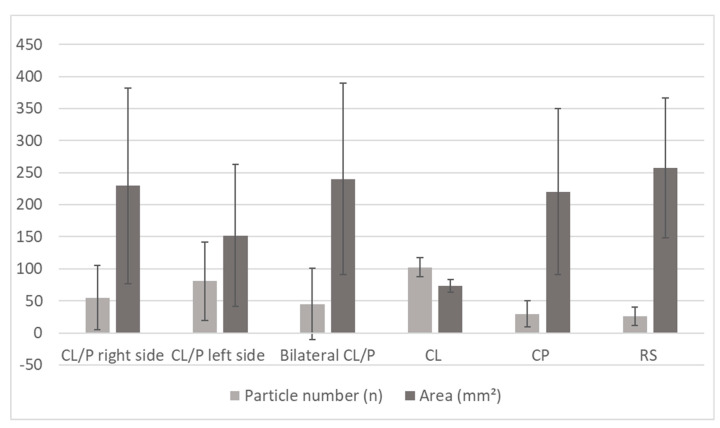
Results of the nine chewing cycles for the different CD cleft formations: right-sided (n = 8), left-sided (n = 18) and bilateral cleft lip and/or palate (n = 6), cleft lip (n = 2), cleft palate (n = 4) and Robin sequence (n = 4). Mean and standard deviation for number of particles (n) and area (mm^2^) are shown. Abbreviations: CL/P = Cleft lip and/or palate; CL = Cleft lip; CP = Cleft palate; RS = Robin sequence.

Furthermore, differences with respect to the cleft formation were observed (Figure 2). Patients with CL exhibited the best masticatory efficiency (n_CL_ = 102.50; A_CL_ = 72.95 mm^2^) among the CD group, followed by those with CP and CL/P left side. The latter exhibited better masticatory efficiency than both right-sided and bilateral CL/P. Meanwhile, RS showed a non-uniform processing of test food due to the big imbalance between the number of particles and area (n_RS_ = 26, A_RS_ = 257.22 mm^2^).

Additionally, the effect of the chewing side (left/right/bilateral) for each of the cleft formations was analyzed. No significant differences among the different formations could found be for the left and bilateral chewing sides, whereas statistically significant differences were recorded for the right chewing side. A significant difference between left CL/P and RS patients was found for the right chewing side, regarding both number of particles (n_CL/P_left_ = 27.06 ± 4.09 vs. n_RS_ = 6.75 ± 8.67; *p* = 0.04) and area (A_CL/P_left_ = 150.39 ± 108.72 mm^2^ vs. A_RS_ = 329.19 ± 117.42 mm^2^; *p* = 0.01). Within the right chewing side too, differences in particle number were observed between left CL/P and CP (n_CP_ = 7.5 ± 8.67), whereas differences in area were found between left and bilateral CL/P (A_CL/P_bilateral_ = 292,65 ± 184 mm^2^, *p* = 0.02), as well as RS and CL (A_CL_ = 92.59 ± 15.78 mm^2^, *p* = 0.03).

With respect to the effect of age on masticatory efficiency (Table 3), statistically significant differences were obtained for the CD group, as older patients with CD (13–21 years) showed a significant better chewing efficiency than younger patients (7–12 years) (n_CD_old_ = 75.36 vs. n_CD_young_ = 46.80; *p* = 0.04). No significant difference induced by age could be found for the healthy group.

Regarding the effect of sex (Table 3), female patients without CD had significantly better masticatory efficiency results compared to healthy male patients (*p* = 0.04), whereas no significant difference was observed in patients with CD.

Referring to the effect of the dentition stage (Table 3), the descriptive analysis of permanent dentition exhibited the best results in the ability of both groups to chew the test bodies. Nonetheless, CD patients had lower results for permanent dentition compared to the control group (n_CD_ = 76.06 vs. n_C_ = 93.67; A_CD_ = 175.87 mm^2^ vs. _AC_ = 143.41 mm^2^). In the first transitional stage, CD patients showed double the amount of chewed parts compared to healthy patients (n_CD_ = 35.75 vs. n_C_ = 17.00), while the surface area was similar (A_CD_ = 262.56 mm^2^ vs. _AC_ = 251.57 mm^2^). An increase in the dentition stage yielded enhanced masticatory efficiency in both groups.

## 4. Discussion

This study aims to determine whether orthodontic patients with CD, such as CL/P and RS, show a different masticatory efficiency compared to healthy ones. In addition, the effect of possible influencing factors, cleft formation, age, sex and stage of dentition, was analyzed. In this study, patients with CD exhibited significantly decreased masticatory efficiency. Therefore, the statistical null hypothesis had to be rejected. The authors are unaware of studies in the current literature dealing with the relationship between CD and masticatory efficiency that have utilized a standardized food model test. Despite this, the results of this study agreed with the study of Zhou et al. in which the masticatory performance of 28 patients with unilateral CL/P with permanent teeth, without orthodontic treatment, was assessed using an advanced light absorption method with peanuts as a test food. The results showed a 54% lower efficiency in patients with CD compared to healthy individuals [27]. In spite of this, the study of Zhou et al. used a different test methodology and a smaller CL/P patient sample size that was not receiving orthodontic treatment, making a comparison between the studies complicated. Moreover, patients with CD showed a significantly reduced ability to masticate on medium-to-hard test bodies in comparison to the healthy group. As the different hardness levels affected the masticatory efficiency of patients with CD, the null hypothesis was rejected. In the literature, the effect of food consistency on masticatory efficiency has been described extensively. Unfortunately, given the diverse study designs and employed methodologies, the comparison of these results with this study is hampered. Despite this, studies suggest that the mastication or processing of harder compounds may enhance both muscle and bone growth, ultimately leading to improved masticatory efficiency [28,29,30]. They also suggest that the ability to process harder foods requires an underlying functional healthy and grown stomatognathic system, which is missing in patients with a CD.

The current study showed that CD participants and their related cleft formations had different masticatory efficiency, and thus the proposed null hypothesis for this matter was also rejected. CL showed the best mastication efficiency, and as the extent of craniofacial deformation increased, the efficiency was found to decrease, showing the worst efficiency in patients with RS. The decreased efficiency in RS could be due to the special dentoskeletal patterns found in the growth and development of patients with a CD [31]. In particular, RS patients not only have a bimaxillary retrognathia with steep mandibular planes, but also have an enlarged gonial angle with a shorter ramus, as well as a shorter maxillar and mandibular sagittal length. Moreover, the intraoral picture of RS shows a malocclusion situation [32,33,34,35,36]. Therefore, not only the occurrence of a cleft, but also the special skeletal pattern, can influence chewing abilities. Regarding patients with CL/P, participants with a unilateral cleft showed better masticatory efficiency than those with a bilateral cleft. This could indicate that the segmentation of the maxilla and the extension of its related defect have a negative impact on mastication efficiency [37,38]. Furthermore, a correlation between the masticatory efficiency of the cleft and non-cleft side was found in this study. The presence of a cleft side may be related to the possible deficiency in development of dental, skeletal and muscle functions, leading to asymmetric growth patterns [39]. This can cause the disruption and imbalance of masticatory muscle formation, and can thus have negative effects such as malocclusion in the stomatognathic system, among others.

Different cleft formations showed statistical significances only in the chewing cycle concerning the right side, and thus the proposed hypothesis was partially rejected. In spite of this, no differences were found for the left chewing side and both chewing side cycles. These differences mainly involved the ability of the left CL/P to chew on the right side in comparison to other groups such as RS, CP and bilateral CL/P. While these may have a rather symmetrical ability to chew on the right and left side, a left CL/P may have caused the overdevelopment of the muscles on the right side in order to compensate for the defect of the left side, therefore making them more developed than in other CDs. Despite this, no similar values for the right CL/P and left side chewing were obtained. While our sample size is appropriate for most comparisons, for RS, the low sample size might have caused a bias in this statistical comparison. Moreover, the sample size for the left side was more than double the right CL/P, which agrees with the prevalence values of the literature that for reasons not yet understood the left side is the most common CL/P [40]. Despite the obtained results, the imbalance of masticatory ability on different chewing sides and CD cleft formations remains to be further studied and tested by other technologies, such as electromyography recordings of the masticatory muscles.

Concerning the effect of secondary factors, such as dentition stage, age and sex, differences between participants with CD and without were observed, and hence the related hypothesis was rejected. In various studies, both dentition stage and age have been reported to have a correlation to the masticatory efficiency [8,41,42,43]. Hame et al. examined the chewing performance of 522 children and 100 young adults using color-changeable chewing gums [42]. In agreement with the present study, they found that increased age und number of functional teeth, as well as a complete permanent dentition were significantly associated with improved masticatory ability. Therefore, it can be assumed that the decay, eruption or loss of a tooth in a mixed dentition negatively impacts the occlusal table. In addition, certain dental conditions, such as loss of a deciduous tooth, could also produce pain during the masticatory process. In this scenario, younger children with mixed dentition could be afraid of pain and avoid the use of the affected teeth. Furthermore, older participants had advanced orthodontic treatment and thus an improved dental arch alignment, which ultimately allows for a better occlusion and enhanced chewing efficiency. Regarding the influence of sex, Zhou et al. showed that there was no difference between patients with CL/P, but that healthy male subjects had better masticatory performance than females [27]. In contrast, in this study, healthy female participants exhibited a superior masticatory efficiency to that of males. Meanwhile, results of the CD group were balanced for both sexes.

The mastication process is the first important step in the digestive process; it is responsible for mechanical food breakdown and the objective of obtaining as many pieces as possible to facilitate the further enzymatic processing [44]. As seen in this study, the ability to break food apart into a higher number of pieces was reduced for patients with CD, which leads to the assumption that the occurrence of a CD can also have negative effects on digestion. In this scenario, special focus on the diet of such patients should be emphasized on an everyday basis, as improved eating habits might lead to the chance to improve their stomatognathic system and their oral health. Moreover, multiple studies have evaluated the effect of specific functional masticatory training on improving the overall oral function and mastication, where positive results were obtained [45,46,47]. For example, Ono et al. confirmed that the use of chewing gums in preschool-aged children increased their biting force by 95% after three months of masticatory training [48]. Functional training, such as tongue and cheek strengthening exercises, can also recover and increase labial closure, skin elasticity, tongue strength and masticatory ability [49,50,51,52]. Therefore, the inclusion of masticatory training programs within interdisciplinary treatment should be encouraged for these patients to help them gain the tools necessary to reduce the influence of an underlying CD on masticatory efficiency.

It is important to recognize some limitations of the study. First, the sample size of certain cleft formations in the CD group was not large enough to obtain reliable statistical analysis. A formal comparison of dentition stages could also not be performed for the same reasons. Despite this, given the low prevalence of these CDs and their specific cleft formations, as well as the monocentric nature of this study, a total of 42 CD patients could still be recruited. While the sample size for the CL/P was higher, a low sample size for RS was obtained. However, given the special skeletal patterns associated with RS, they were not considered within the CL/P group, but rather as a separate one, in order to evaluate the differences among them. Given that the prevalence of RS is even lower than CL/P, a low sample size was obtained. In addition, the employed inclusion criteria were a limiting factor, given that only patients under orthodontic treatment were considered, and orthodontic treatment is known to have an important effect on both the reduction of masticatory and biting abilities [53]. Nonetheless, given the fact that orthodontic treatment may induce the occurrence of pain or discomfort during the masticatory test cycle, and hence reduce masticatory efficiency results, all compared groups within this study were subjected to the same limiting factors and are therefore comparable. Finally, the current study was affected by the COVID-19 pandemic in many ways; routine follow-ups were interrupted, while only emergency cases could be addressed for a period of time, and pandemic regulations directly affected the recruitment of study participants, leading to a lower sample size.

Regardless of the presented limitations, no similar studies to the one presented here exists in the literature yet, exist in the literature, this being the first to evaluate the masticatory efficiency of patients with RS and compare it to that of other CL/P patients, as well as engaging in further analysis of other secondary aspects such as the effect of food hardness, age, sex and dentition stage. In further studies, the influence on masticatory efficiency that results from the different dentoskeletal patterns, malocclusion and orthodontic appliance types throughout the course of orthodontic treatment should be analyzed for these different craniofacial malformations in order to ensure optimal oral and general systemic health in adolescence.

## 5. Conclusions

In this study, the masticatory efficiency of orthodontic patients with CD was evaluated and compared to a healthy group. Within the limitations of this study, the following conclusions can be drawn:

- Patients with CD exhibited significantly decreased masticatory efficiency compared to healthy patients.
- Cleft type or morphology as well as food consistency affected the masticatory function of patients with CD, decreasing with the extension of the cleft defect and the increase in food hardness.
- Differences in right chewing side masticatory efficiency were found between the different CD cleft formations.
- Masticatory efficiency improved with an increase in age and a higher stage of dentition, while no effect of gender was found.

Based on the obtained data, the insertion of a masticatory training program into interdisciplinary treatment is encouraged, with the goal of optimizing treatment outcomes for patients with CD, thereby reducing their oral health burden and achieving a clinical picture closer to that of the healthy population.

## Figures and Tables

**Table 1:** Descriptive evaluation of study participants (n = 119) divided in craniofacial disorder (CD) and control group with respect to age, age groups, sex and stage of dentition. A count of patients and the respective percentage of the population is given for all values, whereas mean and standard deviation (SD) is shown for age (years).

	CD Group			Control Group		
Age (years)	n	%	Mean ± SD	n	%	Mean ± SD
Total	42	100%	13.45 ± 5.20	77	100%	14.3 ± 3.27
7–12 years	20	47.62%	9.95 ± 1.67	22	28.57%	10.45 ± 1.60
13–21 years	22	52.38%	16.64 ± 5.29	55	71.43%	15.84 ± 2.80
Sex	n	%		n	%	
Male	25	59.52%		36	46.75%	
Female	17	40.48%		41	53.25%	
Stage of dentition	n	%		n	%	
Deciduous teeth	0	0.00%		2	2.30%	
First transitional stage	4	9.52%		2	2.30%	
Inter transitional stage	0	0.00%		3	3.90%	
Second transitional stage	20	47.62%		24	31.17%	
Permanent dentition	18	42.86%		46	59.74%	

**Table 2:** Results of masticatory efficiency for patients with craniofacial disorders (CD, n = 42) in comparison to the control group (n = 77) in different chewing cycles. Mean and standard deviation of particle number (n) and area (mm^2^) was given for the different cycles. These are differentiated by the chewing side and employed sample body hardness: total (all nine chewing cycles), right/left/bilateral (three chewing cycles within each hardness level) and soft/medium/hard (three chewing cycles within each chewing side). Statistical significance was considered at *p* < 0.05 and denoted with (*).

Particle Number (n)							
	Total	Right	Left	Bilateral	Soft	Medium	Hard
CD	61.76 ± 10.38	19.83 ± 18.02	20.17 ± 18.84	21.76 ± 3.77	21.43 ± 0.54	21.36 ± 20.24	19.21 ± 3.85
Control	84.58 ± 7.66	24.86 ± 23.46	30.69 ± 26.04	29.18 ± 2.79	27.84 ± 24.44	27.35 ± 22.85	29.40 ± 2.84
*F* ratio	3.13	1.46	5.33	2.50	2.16	2.00	4.54
*p* value	0.08	0.23	0.02 *	0.12	0.14	0.16	0.04 *
**Area (mm^2^)**							
	**Total**	**Right**	**Left**	**Bilateral**	**Soft**	**Medium**	**Hard**
CD	192.91 ± 128.16	192.91 ± 128.16	215.40 ± 19.41	189.91 ± 135.18	199.11 ± 143.23	209.63 ± 20.4	192.53 ± 123.52
Control	146.84 ± 107.12	146.84 ± 107.12	145.40 ± 14.34	151.27 ± 120.24	162.51 ± 129.07	157.53 ± 15.09	151.58 ± 123.24
*F* ratio	4.37	1.52	8.41	2.57	2.02	4.21	3.21
*p* value	0.04 *	0.22	>0.00 *	0.11	0.16	0.04 *	0.08

**Table 3:** Results of masticatory efficiency for patients with craniofacial disorders (CD, n = 42) in comparison to the control group (n = 77). Means and standard deviations are given for both particle number (n) and area (mm^2^), as well as the patient count for each calculation. Results are differentiated by age (7–12 years; 13–18 years), sex and stage of dentition. Statistical significance was considered at *p* < 0.05 and denoted with (*).

	CD Group			Control Group		
	Count	Particle Num. (n)	Area (mm^2^)	Count	Particle Num. (n)	Area (mm^2^)
Age						
7–12 years	22	46.80 ± 11.79	207.42 ± 28.84	22	80.45 ± 49.91	132.23 ± 99.37
13–18 years	20	75.36 ± 11.24	179.72 ± 132.60	55	86.25 ± 81.29	152.69 ± 110.40
*p*		0.04 *	0.49		0.35	0.43
Sex						
Male	25	61.96 ± 53.90	181.29 ± 25.79	36	67.44 ± 55.09	168.61 ± 115.03
Female	17	61.47 ± 55.88	209.99 ± 138.50	41	99.66 ± 84.20	127.73 ± 97.07
*p*		0.98	0.48		0.04 *	0.04 *
Stage of dentition						
Deciduous teeth	0	/	/	2	94.50 ± 54.45	82.10 ± 31.27
First transitional stage	4	35.75 ± 33.38	262.56 ± 182.15	2	17.00 ± 1.41	251.57 ± 12.39
Inter transitional stage	0	/	/	3	57.67 ± 57.84	181.77 ± 142.58
Second transitional stage	20	54.10 ± 43.32	194.32 ± 120.56	24	75.38 ± 54.73	145.73 ± 104.75
Permanent dentition	18	76.06 ± 65.59	175.87 ± 126.73	46	93.67 ± 83.50	143.41 ± 109.88

## Data Availability

Data are not available due to data protection of patients.
